# 

**Published:** 1862-07

**Authors:** 


### WORKS PUBLISHED DURING THE QUARTER.

### 1. *Medicine.*

BELCHER.—Reformatories for Drunkards. By T. W. Belcher, B.M., M.A. 8vo, sewed, 8d.

BENNETT.—Introduction to Clinical Medicine. Being Lectures on the Method of Examining Patients, and the means necessary for arriving at an exact Diagnosis. By J. Hughes Bennett, M.D. Fourth Edition, sm. post 8vo, cloth, 5s.

BAKER BROWN.—On Ovarian Dropsy: its Nature, Diagnosis, and Treatment, the Result of Thirty Years' Experience. By I. Baker Brown, F.R.C.S. (by Exam.) Post 8vo, cloth, 7s.

EARLE.—The Mammary Signs of Pregnancy and of Recent Delivery. By J. Lumley Earle, M.D. 8vo, cloth limp, with Plates, 2s. 6d.

EDWARDS.—The Examination of the Chest, in a Series of Tables. By George N. Edwards, M.D. Cantab. Royal 8vo, cloth, 2s. 6d.

HABERSHON.—On Diseases of the Abdomen, Stomach, and other Parts of the

Alimentary Canal. By S. O. Habershon, M.D., F.R.C.P. Second Edition, considerably Enlarged, 8vo, cloth, 14s.

Jones.—On the Use of Perchloride of Iron and other Chalybeate Salts in the Treatment of Consumption: a Clinical Inquiry into their Physiological Action and Therapeutic Properties. With a Chapter on Hygiene. By James Jones, M.D. Lond., M.R.C.P. Crown 8vo, cloth, 3s. 6d.

Leudet.—Clinical Essay on the Mineral Waters of Eaux Bonnes (Pyrenees) and their Value in Consumptive Diseases. By Dr. Lucien Leudet. 8vo, sewed, 1s.

Martyn.—Hospitals Past and Present. By Samuel Martyn, M.D., M.R.C.P. 8vo, 1s.

Meadows.—A Manual of Midwifery. By Alfred Meadows, M.D., M.R.C.P. 32mo, cloth, 3s. 6d.

Obstetrical Society.—Transactions, Vol. III., for the Year 1861. 8vo, cloth, 15s.

Pavy.—Diabetes, Researches on, its Nature and Treatment. By F. W. Pavy, M.D., F.R.C.P. 8vo, cloth 8s. 6d.

Ryan.—Infanticide: its Law, Prevalence, Prevention and History. By William Burke Ryan, M.D. Lond., F.R.C.S. Eng. 8vo, cloth, 5s.

Smith.—Consumption: its Early and Remediable Stages. By Edward Smith, M.D., L.L.B. Post 8vo, cloth, 10s. 6d.

Tilt.—On Uterine and Ovarian Inflammation, and on the Physiology and Diseases of Menstruation. By E. J. Tilt, M.D., M.R.C.P. Third Edition, with Illustrations, 8vo, cloth, 12s.

Townley.—Parturition without Pain. By James Townley, L.R.C.P. Edin., F.L.S. Post 8vo, cloth, 2s. 6d.

Wells.—On Long, Short, and Weak Sight, and their Treatment by the Scientific Use of Spectacles. By J. Soelberg Wells. With Engravings on Wood and Stone, 8vo, cloth, 5s.

### 2. *Surgery.*

Acton.—The Functions and Disorders of the Reproductive Organs in Childhood, Youth, Adult Age, and Advanced Life, considered in their Physiological, Social, and Moral Relations. By William Acton, M.R.C.S. Third Edition, entirely re-written, 8vo, cloth, 10s. 6d.

Ashton.—Prolapsus, Fistula in Ano, and Hæmorrhoidal Affections; their Pathology and Treatment. By T. J. Ashton. Post 8vo, cloth, 2s. 6d.

Bigg.—Spinal Curvature: the Mechanical Appliances adapted for its Successful Treatment. By Henry Heather Bigg, Assoc. Inst. C.E. With Engravings, post 8vo, cloth, 4s. 6d.

Chance.—On the Nature, Causes, Variety and Treatment of Bodily Deformities, in a Series of Lectures delivered at the City Orthopœdic Hospital. By E. J. Chance, F.R.C.S.E. Part I., post 8vo, cloth. 12s. 6d.

Heath.—A Manual of Minor Surgery and Bandaging, for the Use of House-Surgeons, Dressers, and Junior Practitioners. By Christopher Heath, F.R.C.S. Second Edition, Enlarged, with Illustrations, fcap. 8vo, cloth, 5s.

Milton.—On the Treatment of Gonorrhœa without Specifics. By J. L. Milton, M.R.C.S. Eng. Second Edition, 8vo, cloth, 5s.

Smith.—Hæmorrhoids and Prolapsus of the Rectum, their Pathology and Treatment, with especial reference to the Use of Nitric Acid; with a Chapter on the Painful Ulcer of the Rectum. By Henry Smith, F.R.C.S. Third Edition, fcap. 8vo, cloth, 3s.

